# Sulfur isotopes ratio of atmospheric carbonyl sulfide constrains its sources

**DOI:** 10.1038/s41598-018-37131-3

**Published:** 2019-01-24

**Authors:** Alon Angert, Ward Said-Ahmad, Chen Davidson, Alon Amrani

**Affiliations:** The Institute of Earth Sciences, The Hebrew University of Jerusalem, Jerusalem, Israel

## Abstract

Carbonyl sulfide (COS) is the major long-lived sulfur bearing gas in the atmosphere, and is used to estimate the rates of regional and global (both past and current) photosynthesis. Sulfur isotope measurements (^34^S/^32^S ratio, δ^34^S) of COS may offer a way for improved determinations of atmospheric COS sources. However, measuring the COS δ^34^S at the atmospheric concentrations of ~0.5 ppb is challenging. Here we present high-accuracy δ^34^S measurements of atmospheric COS done by gas chromatograph (GC) connected to a multicollector inductively coupled plasma mass spectrometer (MC-ICPMS), after pre-concentrating from 2-liters of air. We showed that the precision of COS δ^34^S measurement for gas standards is ≤0.2‰, and that N_2_ and CO_2_ in the gas standard mixture had no effect on the measured δ^34^S. Natural air samples were collected in Israel and in the Canary Islands. The COS δ^34^S values in both locations were found to be 13.2 ± 0.6‰, and are believed to represent the background tropospheric value. This δ^34^S value is markedly different from the previously reported value of 4.9‰. We estimate the expected isotopic signature of COS sources and sinks, and use the δ^34^S value of atmospheric COS we measured to estimate that ~48% of it originates from the ocean.

## Introduction

The atmosphere contains about 0.5 ppb carbonyl sulfide (COS), with a lifetime of few years^1^. Because it is relatively long-lived, the COS is hypothesized to be a large source of background stratospheric aerosols which have important control on Earth albedo and stratospheric chemistry, including the ozone layer^2,3^. In addition, since COS follows a similar pathway to CO_2_ through stomata during photosynthesis, it can be used to estimate the photosynthesis rates, mainly on a global scale for both the present and for the last century^4–7^, but also on regional scale^8^. The main natural source of COS to the atmosphere is the ocean, both as direct COS emission, and indirect as carbon disulfide (CS_2_) and dimetheylsulfide (DMS) emissions that rapidly oxidized to COS^9^. Anthropogenic sources of COS are dominated by indirect sources (CS_2_) and include rayon production, aluminum production, coal combustion, biomass burning, oil refineries and fuel combustion^10,11^.

The relatively small decadal trends in COS atmospheric concentrations indicate that on a global scale, the sources and sinks are approximately balanced. A recent review of the atmosphere COS budget^12^ highlights the major knowledge gaps. Previous studies also demonstrated the large uncertainty in the global COS budget, with 3-fold uncertainty in plant uptake^4^ and up to 8-fold uncertainty in the ocean source^13^ and 3-fold uncertainty in the anthropogenic COS sources^5^. The uncertainties in ocean COS emissions are related in part to the physicochemical and biogeochemical models that are used for these estimations, and the scarcity of direct measurements^13,14^.

Sulfur isotope measurements (^34^S/^32^S ratio, δ^34^S) of COS are suggested here as a novel approach for the determinations of atmospheric COS sources. The isotopic approach assumes that COS sources (mainly oceanic and anthropogenic, but also biomass burning and soil) have distinct δ^34^S values. Therefore, the contribution of each COS source to the atmosphere can be calculated using the appropriate isotope mass balance equations, and knowledge on the fractionation during uptake by the sinks (mainly plants, but also atmospheric oxidation and soils). The isotopic mass balance, assuming steady state since the long-term trends are small^7^, can be presented by the following equation:

1 $$\begin{array}{c}{\rm{O}}\,\times \,{{\rm{\delta }}}^{{\rm{34}}}{{\rm{S}}}_{\mathrm{COS} \mbox{-} {\rm{ocean}}}+{\rm{A}}\,\times \,{{\rm{\delta }}}^{{\rm{34}}}{{\rm{S}}}_{\mathrm{COS} \mbox{-} {\rm{anthropogenic}}}={\rm{P}}\,\times \,{({\rm{\delta }}}^{{\rm{34}}}{{\rm{S}}}_{\mathrm{COS} \mbox{-} {\rm{atmosphere}}}+{{\rm{\varepsilon }}}_{{\rm{p}}})\\ +{\rm{S}}\,\times \,{({\rm{\delta }}}^{{\rm{34}}}{{\rm{S}}}_{\mathrm{COS} \mbox{-} \mathrm{atmosphere}}+{{\rm{\varepsilon }}}_{{\rm{s}}})+{\rm{X}}\,\times \,{({\rm{\delta }}}^{{\rm{34}}}{{\rm{S}}}_{\mathrm{COS} \mbox{-} \mathrm{atmosphere}}+{{\rm{\varepsilon }}}_{{\rm{x}}})\end{array}$$

The left side of the equation represents the sources and their isotopic composition: O is the flux from the ocean (combined direct and indirect fluxes), δ^34^S_COS-ocean_ is the average weighted ocean source isotopic composition, A is the anthropogenic flux, and δ^34^S_COS-anthropogenic_ is its average isotopic composition. The right side represents the sinks, where P, S, and X are the sinks by plant, soil, and atmospheric oxidation, respectively. The possible fractionations during uptake are represented by ε with the corresponding subscript. Estimating the relative contributions of the sources to atmospheric COS will provide an important constraint to the COS budget and photosynthesis models, and thus reduce their uncertainties.

Measurements of sulfur isotopes in atmospheric COS are challenging because of its low concentrations: ~0.5 ppb. A recent method of COS δ^34^S analysis on fragments ions using a pre-concentration air system coupled with isotope ratio mass spectrometer (IRMS) requires 10’s nmol which translates to hundreds of liters of air per analysis^15^. With this method these researchers were able to provide a single δ^34^S value of COS of a compressed air sample from one location in Japan (Kawasaki). However, the need for hundreds of liters of air per analysis still limits the applicability of this method.

A more tractable analytical approach for the analysis of trace atmospheric sulfur compounds is the coupling of a gas chromatograph (GC) with a multicollector inductively coupled plasma mass spectrometer (MC-ICPMS)^16^. This method enables the measurements of δ^34^S values in individual volatile and gas compounds and requires a sample size of the pmol level or about 10^4^-fold lower than that of a regular IRMS^16–18^. The current sensitivity of the GC/MC-ICPMS required only 1–2 L of air for reliable δ^34^S analysis of atmospheric COS. Here we use the GC/MC-ICPMS for accurate and precise δ^34^S determination of COS in low volumes of atmospheric air in two locations.

## Results

### Precision and accuracy of COS δ^34^S analysis at low concentrations

The method we used was a combination and modification of two existing methods. One is the Tenax resin pre-concentration of COS from air^15,19^ and the second is the δ^34^S analysis of gases using GC/MC-ICPMS^18^. To ensure that our method preserves the original δ^34^S value of the measured COS, we have measured the following COS standards using two introduction methods, direct injection and pre-concentration. These gas mixtures were calibrated against Mix 1 that contained several sulfur compounds including COS at concentration of ~21 ppm. The COS main standard (4.7% in He, hereafter “Mix 2”) was diluted and mixed with other gases to make additional 2 mixtures: a 5.2 ppm of COS in He (99.995% pure) hereafter “Mix 3”, and 1.7 ppb COS, diluted in ~500 ppm CO_2_ and N_2_ as balance, hereafter “Mix 4”. The N_2_ was added to the mixture to verify successful capture of COS on Tenax when diluted by the main component of the atmosphere. CO_2_ was added since it condenses in the Tenax trap temperature (−90 °C), and thus can potentially interfere. Note that both N_2_ and O_2_ do not spontaneously react with COS, nor will it react with the Tenax at this temperature. Other gases are not expected to interfere in COS trapping on Tenax because they are in trace amounts in the atmosphere, and even if captured by the Tenax, they will be separated by the GC column.

**Figure 1:**
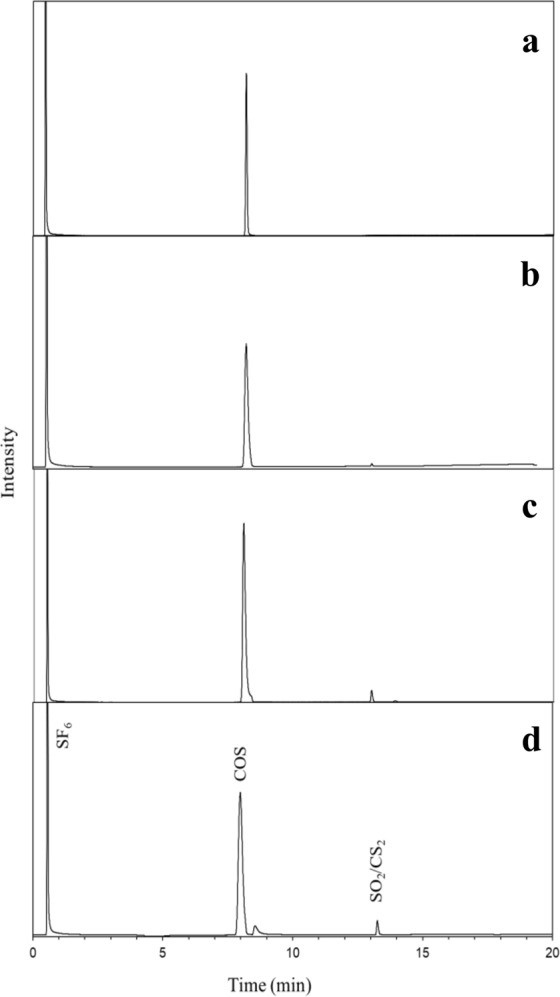
Chromatogram produced by the GC/MC-ICPMS system for the injection of (**a**). 5.2 ppm COS standard by direct injection (**b**). 5.2 ppm COS standard by pre-concentration system (**c**). 1.7 ppb COS standard by pre-concentration system (**d**). Air sample by the pre-concentration system. The SF_6_ peaks are used as internal standards in each chromatogram and are calibrated every 3–4 samples by known standards as detailed in the Methods section.

Standards mixtures 2–4 have the same original COS gas which was isotopically calibrated against our laboratory standards. The first standard (Mix 2) was measured directly, without the pre-concentration system. The second standard (Mix 3) was measured both directly and by the pre-concentration system. And Mix 4 was measured only by the pre-concentration system (Fig. 1, Table 1). Hence, measuring these standards tests for possible fractionation during pre-concentration, and for possible interference by N_2_ and CO_2_ during capture on the Tenax resin. Table 1 summarize the results of these tests. The results show that there is no fractionation involved with the pre-concentration step, even when N_2_ and CO_2_ are present. In addition, for the Mix 3 standard that was measured both by pre-concentration and by direct injection to the GC, we found that the yield of the pre-concentration system is better than 97%.

**Table 1:** Results of GC/MC-ICPMS analysis of COS standards.

Standard	Balance	Conc.	Method	Amount measured	Amount RSD error	δ^34^S	Error	Repetitions
mix #	gas	(mol/mol)		pmol	%	‰	std	n
2	He	4.70%	direct injection^1^	265	1.7	−6.2	0.1	5
3	He	5.2 ppm	direct injection	20	2.8	−6.0	0.2	17
3	He	5.2 ppm	pre-conc.	20	2.0	−6.1	0.0	3
4	N_2_+CO_2_	1.7 ppb	pre-conc.	65	3.0	−6.0	0.1	4

### Analysis of natural air samples

Air was sampled in Jerusalem, Israel (31°46′12″N/35°11′51″E) in August and October 2017 and March 2018, and in the Canary Island of Fuerteventura (28°43′30″N/13°50′33″W) in February 2018. To check the stability and precision of air measurements of COS in air samples using Sulfinert 2.25 L cylinders (see Methods section), we have measured 9 air samples next to the institute of Earth Sciences in the Hebrew University, Jerusalem, during August and October 2017. The results are presented in Table 2 (all the δ^34^S values in this work are reported against VCDT). The average δ^34^S value for these measurements set was 13.4 ± 0.5‰ (std, 1σ), which demonstrates the stability and precision of the system over time. The higher errors compared to the standard measurements are most probably introduced by COS blanks left in the sampling cylinders after the cleaning procedure. It is possible that an improved cleaning procedure (e.g. repeating all the steps twice) will lower this blank. The RSD (Relative Standard Deviation) of the peak area is 10.4%, which represents the variability in the MC-ICPMS sensitivity, which was not corrected against a COS concertation standard in this first campaign. We have performed two more sampling campaigns using a different method of sampling (electropolished canisters, see Methods) during February and March 2018 to check for a possible difference in COS δ^34^S values that arise from geographic location. Table 2 details the location, COS concentration and isotopic composition of each sampling campaign. There was no apparent difference in δ^34^S values between samples taken in Sulfinert treated stainless steel cylinders, and those taken with electropolished stainless steel canisters, indicating that both are acceptable options for COS sampling. The measurements of air sampled resulted in an average (±1σ std) concentration of 0.52 ± 0.01 ppb for the two sites. This concertation agrees well with the known concertation of COS in the atmosphere, and thus indicate good preservation of the samples during the few weeks from sampling to analysis. The average δ^34^S value (±1σ std) for the February-March campaign was found to be 12.8 ± 0.5‰ (n = 3) for Jerusalem, and 13.1 ± 0.7‰ (n = 3) for the Canary Islands. The overall average for all months and both location is 13.2 ± 0.6‰ (with no significant temporal or spatial variation).

**Table 2:** Results of GC/MC-ICPMS analysis of COS in air samples from Israel and the Canary Island.

Air sample	Amount pmol	Conc. ppt	Conc. error	δ^34^S ‰	δ^34^S error	Repetitions
			RSD%		Std ‰	n
Israel 1	28	—	10.4	13.4	0.5	9
Israel 2	38	502	6.6	12.8	0.5	3
Canary Islands	45	533	7.5	13.1	0.7	3

**Israel 1** –samples were taken by Sulfinert cylinders in Israel at the Institute of Earth Science, The Hebrew University of Jerusalem (31°46′12″N/35°11′52″E).

**Israel 2 -** samples were taken by electropolished canisters in Israel at the Institute of Earth Science, The Hebrew University of Jerusalem (31°46′12″N/35°11′52″E).

**Canary Islands -** samples were taken by electropolished canisters in the Canary Islands at Punta de Tivas, Fuerteventura island (28°43′30″N/13°50′33″W).

## Discussion

Our COS standards analysis showed that the method we used is highly useful and applicable for measurements of COS δ^34^S values at atmospheric concentrations. There were no apparent effects of the pre-concentration step on the precision and accuracy, and no interfering effects from other gases in the gas matrix used (either He or N_2_ and CO_2_). These results provide confidence that the measurements of natural air samples represent reliably the atmospheric COS δ^34^S values.

**Figure 2:**
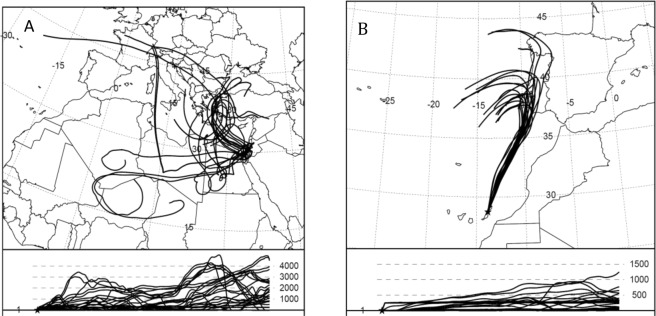
Back trajectories for 10 days (produced by NOAA HYSPLIT MODEL^20^) of the air before arriving to the air sampling locations during Feburary and March 2018: (**A**) Israel, (**B**) Canary Islands.

The natural air samples showed an average δ^34^S value of 13.2 ± 0.6‰, with no detectable variation in isotopic composition between the Canary Islands and Israel, despite a very different trajectory of the air before arriving to the sampling locations. A back-trajectory analysis by NOAA’s HYSPLIT^20^ shows that the history of the air sampled in the Canary Islands was mostly of a path along the north Atlantic, which only slightly brushed against the western edge of Europe (mostly Portugal), before continuing over the Atlantic to the sampling point. In contrast, the air sampled in Israel had a much more continental path (Fig. 2). The similar values for Israel and the Canary Islands are probably the result of the long life time of COS in the atmosphere, which is a few years^1^. Hence, this similarity seems to indicate that the δ^34^S value we measured represents the clean atmosphere.

The only previously published measurement of atmospheric COS^15^ reported a measurement of a single sample with δ^34^S value of 4.9 ± 0.3‰. This value is much lighter from an estimated^21^ value of 11‰, and from the value we measured (13.2 ± 0.6‰) in two independent locations. This mismatch may stem from the fact that Hattori *et al*.^15^ single δ^34^S value was measured from compressed air that was collected at the manufacturer’s factory in Kawasaki, Japan, and might not represent the clean atmosphere COS signal. It might also result from the markedly different methods used in the two studies. More specifically, the need to pre-concentrate hundreds of liters of air in Hattori *et al*.^15^ as well as the analysis on fragments ions may introduce additional sources of error. However, although it seems unlikely, we cannot rule out at this stage the possibility that the δ^34^S value of COS is not homogenous globally and so there are real and significant difference between the δ^34^S values between Japan and Israel/Canary Islands. Further δ^34^S analyses of COS from around the world are needed to confirm that.

Assuming that the atmosphere is well mixed (homogenous) in regards to COS, and using our clean air atmospheric COS δ^34^S value (i.e. 13.2 ± 0.6‰) to represent it, there are several important implications that can be drawn in relation to the contribution of COS to background aerosols and to the relative sources of COS to the atmosphere.

COS is suggested to be an important source of sulfur to the stratosphere background aerosols^1,2^. Based on the range of expected fractionation for COS oxidation at the tropopause and the stratosphere (ε_x_ = −8‰ to −2.3‰^22^), and the atmospheric δ^34^S value we measured, the COS oxidation products (which end up as stratospheric aerosols) are expected to have an isotopic value of 4.9–10.6‰ (δ^34^S_products_ = δ^34^S_COS_ + ε_x_). Given the measured δ^34^S of stratospheric background aerosols^23^ of 2.6‰, our COS isotopic measurements are consistent with COS being an important, but not the single, source for these aerosols.

**Figure 3:**
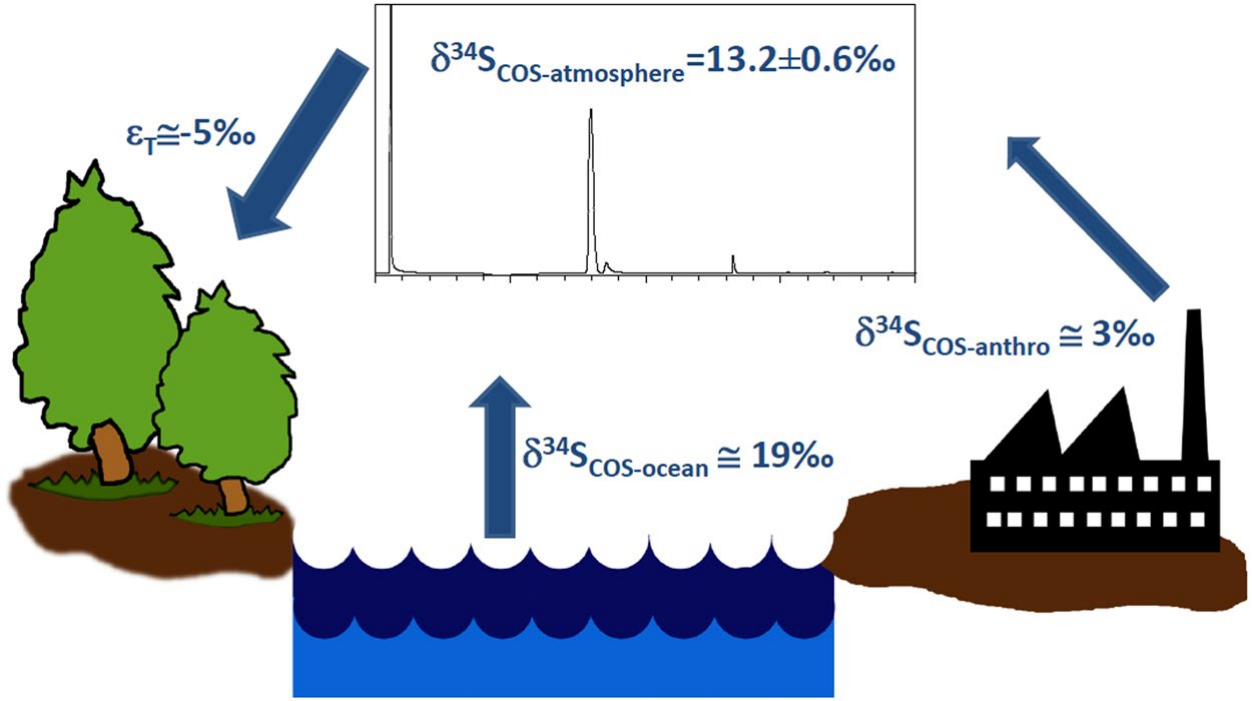
A scheme illustrating the isotopic mass balance for atmospheric COS, assuming values for anthropogenic emissions (δ^34^S_COS-anthro_), for ocean emissions (δ^34^S_COS-ocean_), and for fractionation during (plant dominated) uptake (ε_T_). Using the COS δ^34^S value we measured in the atmosphere (appears in a GC/MC-ICPMS chromatogram) it is estimated by Equation 2 that ~48% of COS emissions originate in the ocean. (Images drawn by Ayelet Angert).

It is also possible to constrain the relative contribution of the ocean and anthropogenic COS sources, by a simple 1-box isotopic balance model as illustrated in Fig. 3. For this model we use the following initial assumptions regarding the isotopic signatures of COS sources and sinks.

Ocean source: We assume here that the isotopic composition of COS emitted from the ocean is ~19‰, with negligible fractionation during degassing, similar to the values recently found for another oceanic trace sulfur compound - DMS^24^. This assumption is feasible since both COS and DMS are degradation products of organic sulfur. Organic sulfur of marine microorganisms is produced by microbial assimilatory sulfate reduction process from seawater sulfate (+21.1‰) with a small (<3‰) fractionation^25^, similar to that found for DMSP/DMS^24,26,27^. Moreover, part of the oceanic flux of the COS is the result of oxidation of DMS to COS^14^, and if no fractionation is involved in the oxidation then the DMS and COS are expected to have similar δ^34^S value.

Anthropogenic source: The δ^34^S values of sulfate and sulfur dioxide related to fossil fuel combustion and vehicle exhausts are reported to be in the range of +4‰ to +8‰, while refineries in Washington, USA and Michigan, USA were found to have a δ^34^S value of −1.6% and +2‰ to +4‰ respectively^28^. Sulfate aerosols above heavily industrialized areas in northern America and Europe were found^29–31^ to have δ^34^S values between 3‰ to 9‰. According to the values above, it is estimated^28^ that anthropogenic sulfate have average δ^34^S value of 3 ± 2‰^28^. If COS is released to the atmosphere from its anthropogenic sources with no fractionation or a very small one, then we can assume similar COS δ^34^S values as reflected in sulfate aerosols of industrial sulfate (~3‰).

Plant uptake: Previous studies^32,33^ have shown that most of the COS that diffuses into plant leaves is hydrolyzed immediately, and that the back-diffusion is negligible. In such case, the overall fractionation of plant uptake is that of the fractionation in diffusion, and possible fractionation during enzyme-mediated fractionation will have no effect. We calculate that the expected fractionation in binary diffusion of COS in N_2_, according to the theory of binary diffusion of gases^34^, is ~−5‰.

Atmospheric oxidation, and soil uptake: The overall fractionation in all atmospheric oxidation processes is estimated to be relatively small, −8‰ in the tropopause and lower in the stratosphere^22^. It is also estimated that less than 10% of the COS transported to the stratosphere is consumed there, while the rest returns to the troposphere^1^. The fractionation in uptake by few soil bacteria genera was found to be in the range of −2‰ to −4‰, and genus dependent^35^.

Since the rates of soil uptake and atmospheric oxidation are small relative to plant uptake, and since the fractionations in these processes are not well constrained, we will simplify Equation 1 by pooling together all the sinks with one overall fractionation (ε_T_) that will be assumed to be −5‰. Assuming mass balance we get:

2 $${\rm{O}}/{\rm{A}}=-\,{({\rm{\delta }}}^{{\rm{34}}}{{\rm{S}}}_{\mathrm{COS} \mbox{-} {\rm{anthropogenic}}}-{{\rm{\delta }}}^{{\rm{34}}}{{\rm{S}}}_{\mathrm{COS} \mbox{-} \mathrm{atmosphere}}-{{\rm{\varepsilon }}}_{{\rm{T}}}{)/({\rm{\delta }}}^{{\rm{34}}}{{\rm{S}}}_{\mathrm{COS} \mbox{-} \mathrm{ocean}}-{{\rm{\delta }}}^{{\rm{34}}}{{\rm{S}}}_{\mathrm{COS} \mbox{-} \mathrm{atmosphere}}-{{\rm{\varepsilon }}}_{{\rm{T}}}),$$

where O/A is the ratio between the ocean and anthropogenic sources.

Using our own measured δ^34^S value for Jerusalem and the Canary Islands of 13.2‰, it is estimated (Fig. 3) that about half of the atmospheric COS (48%) comes from the ocean, while the rest is contributed by anthropogenic emissions. This initial estimate (based on our measurements) is indeed in broad agreement with previous estimates^4,5,14^. In contrast, using the only previously published measurement of atmospheric COS δ^34^S of 4.9 ± 0.3‰^15^ gives an O/A ratio of 16% (i.e. 84% of the COS source is anthropogenic), which is far from all current estimates.

The discussion above and the simple model calculations we made show that the isotopic approach for COS sources attribution is feasible and promising. However, it is possible that deviations for the simplifying assumptions above are non-negligible. To improve this approach, there is a need for direct measurements of the sources and sinks isotopic signatures, and a full scale atmospheric sampling plan to reveal variations in both space (e.g. down-wind of major rayon production areas) and time (e.g. increase in δ^34^S resulting from summer photosynthetic drawdown). Results from such measurements could be then analyzed by a transport model, that will allow to separate the contributions of the different sources.

## Methods

### Air sampling and trapping

**Figure 4:**
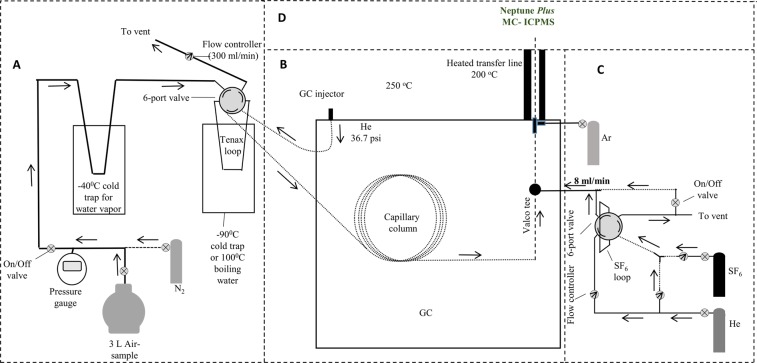
Schematic layout of the analytical system: (**A**) COS pre-concentration system, (**B**) gas chromatograph (GC), (**C**) SF_6_ standard injection system, (**D**) The Neptune plus multi-collector inductively coupled plasma mass spectrometry (MC-ICPMS) system.

Two slightly different methods were used. In the initial method (samples from August and October 2017) we used evacuated 2.25 L Sulfinert treated stainless steel cylinder (High Pressure Sample Cylinder, Restek) for air sampling. The cylinders were equipped with two Swagelok valves, one on each side. In order to clean the cylinders, we heated them to 60 °C with a constant He (99.995%) flow of 100 ml min^−1^ for several hours. Before air sampling the He in the canisters was analyzed to make sure that the background of absorbed COS in the canister is not larger than 2 pmol. For δ^34^S analysis of air in the canisters, we used a constant He flow of 100 ml min^−1^ for 30 minutes through the cylinder and into the pre-concentration system, described below in this section. In this preliminary method we did not accurately estimate the percent of the air sample that was extracted from the cylinder and therefore we did not present concentrations for this analysis. In the Updated Method (February and March 2018), instead of the 2.25 L sample cylinder, we used much lighter electropolished stainless steel 3 L canisters (To-can, Restek), which allow easier shipping. Previous work^7^ have shown that COS is stable over weeks during storage in such stainless-steel electropolished canisters, even if water-vapor is present. Hence, water vapor was not removed during sampling. We also added a pressure gauge (0.25% precision, Ashcroft) for accurate measurement of air sample extraction. In order to clean these canisters, they were vacuumed to a pressure of ~2 Kpa and then filled with N_2_ (99.99%, pre-checked to be COS free) up to 92 Kpa, before adding 2 ml of purified water. The canisters were then pumped down to ~30 Kpa and heated to 120 °C for 1 hour. Then they ware vacuumed and filled with N_2_ repeatedly 10 times. Before air sampling, the N_2_ in the canisters was measured to make sure that the background of absorbed COS in the canister is not larger than 2 pmol (~5% of typical atmospheric COS sample). The error that these blanks can introduce is less than 0.6‰, based on the blanks isotopic composition. After air sampling, the canisters were pressurized with N_2_ (99.99) to 350 Kpa, and this pressure was utilized to extract 67% (~2 L) of the air sample into the pre-concertation system (Fig. 4A). This system, which is similar but simpler than that used by Hattori^15^, collects the COS from the gas stream by a 1.59 mm (ID) × 3.18 mm (OD) Teflon tube trap filled with 50 mg Tenax (TA, 60–80 mesh; Sigma-Alorich (MO, USA)) cooled by ethanol at −90 °C. A flow controller keeps the flow below 300 ml min^−1^. Before the Tenax trap, a cold trap cooled by ethanol at −40 °C is used to remove water vapor. The Tenax trap is connected through a six-way valve to a GC. After 65 min (with decreasing flow rates), the pressure in the cylinder dropped down to ~115 Kpa. The Tenax trap is then warmed by hot (boiling) water to ~100 °C, and the six-way valve is used to inject the sample to the GC. In contrast to Hattori *et al*.^15^ no pre-concentration in liquid N_2_ trap before the GC was needed.

### Instrumentation

The system employed for the S-isotope analysis of COS consisted of a gas chromatograph (GC, Trace 2000 series, Thermo, Germany) coupled with a Neptune Plus™ MC-ICPMS (Thermo Scientific, Bremen, Germany) as described in Fig. 4. The GC was equipped with a split/splitless injector for direct injection of volatile samples and a heated (70 °C) six-way valve gas inlet system (Valco Instrument Co, TX, USA) for the introduction of gaseous compounds with a computer-controlled actuator. The GC column (60 m * 0.320 mm, GS-GASPRO, Agilent Technologies) is able to separate cleanly between SO_2_ or CS_2_ and COS. A transfer line, heated to 200 °C, connected the GC to the plasma source^18^.

The S species were then atomized and ionized in the plasma source and yielded ^32^S^+^ and ^34^S^+^ ions that were transferred to the mass spectrometer unit of the GC/MC-ICPMS system for isotope ratio analysis. The Neptune MC-ICPMS system is a double-focusing magnetic-sector instrument equipped with eight moveable Faraday detectors and one fixed detector for simultaneous detection of different masses. The Faraday detectors were positioned to simultaneously collect ^32^S^+^ and ^34^S^+^. Table 1 presents the operational conditions of the GC-MC-ICPMS system. Data processing procedure was as described in detail elsewhere^16,18^.

### Reagents and standards

DMS (>99%), Thiophene (99+%) and carbon disulfide (CS_2_, anhydrous ≥99%) were purchased from Sigma-Aldrich (MO, USA). Sulfur hexafluoride (SF6, 500 ppm in helium) was purchased from Praxair (PA, USA). A standard for S compounds in He, ~21ppm (20.8 ppm COS, 20.5 ppm CS_2_, 20.9 ppm DMS, 20.9 ppm Ethyl thiol, 20.7 ppm H_2_S, 20.8 ppm Methyl thiol) was purchased from Air Liquide America (PA, USA) (“Mix 1”). A COS gas mixture (4.7%) in helium as balance gas (“Mix 2”) was purchased from Air Liquide America. The sulfur isotope reference materials NBS-127 (BaSO4; δ^34^S = 21.1‰), IAEA-S-1 (Ag_2_S; −0.3‰), and IAEA-SO-6 (BaSO_4_; −34.1‰) were purchased from the National Institute of Standards and Technology (NIST, USA) and were used for calibration of all the in-house standards.

The δ^34^S values of Mix 1 were calibrated against in-house liquid standards DMS (−3.0 ± 0.1‰), CS_2_ (17.2 ± 0.1‰) and Thiophene (9.6 ± 0.2‰) (“Mix 5”) that were pre-calibrated against international standards (using elemental analyzer isotope ratio^16,18^. These standards, diluted in toluene to form ~81 pmol μL^−1^, were injected directly in to the GC injector (1 µl, split 5, ~16 pmol on column) as detailed in Said-Ahmad *et al*.^18^. Then, in each day of analysis (COS standards of air) both Mix 1 and Mix 5 were injected to calibrate the internal standard SF_6_. All the δ^34^S values in this work are reported against VCDT.

## Data Availability

All data generated or analysed during this study are included in this published article.
